# Anthropometric risk factors for ovarian cancer in the NIH-AARP Diet and Health Study

**DOI:** 10.1007/s10552-020-01377-y

**Published:** 2021-01-22

**Authors:** Sebastian E. Baumeister, Inga Schlecht, Britton Trabert, Michael Nolde, Christa Meisinger, Michael F. Leitzmann

**Affiliations:** 1 Chair of Epidemiology, Ludwig-Maximilians-Universität München, UNIKA-T Augsburg, Neusässer Str. 47, 86156 Augsburg, Germany; 2 Independent Research Group Clinical Epidemiology, Helmholtz Zentrum München, German Research Center for Environmental Health, Neuherberg, Germany; 3 University of Münster, Münster, Germany; 4 Department of Epidemiology and Preventive Medicine, University of Regensburg, Regensburg, Germany; 5 Division of Cancer Epidemiology and Genetics, National Cancer Institute, National Institutes of Health, Bethesda, MD USA

**Keywords:** Adiposity, Body fat, Obesity, Ovarian cancer risk

## Abstract

**Objective:**

Identifying potentially modifiable risk factors for ovarian cancer is essential for prevention because this cancer is predominantly detected at a late stage. Here, we estimated the relations of general adiposity and measures reflecting body fat distribution to the risk of epithelial ovarian cancer.

**Methods:**

We ascertained 683 ovarian epithelial cancers (343 high-grade serous, 141 non-high grade serous) among 145,575 women, aged 50–72 years (median follow-up 12.6 years), from the National Institutes of Health—American Association of Retired Persons (NIH-AARP) Diet and Health Study. Using Cox models, we estimated confounder-adjusted hazard ratios (HRs) and 95% confidence intervals (CI) for associations of overall ovarian cancer, high-grade serous and non-high-grade serous carcinoma with body mass index, waist circumference, hip circumference, waist–hip ratio, waist–height ratio, body adiposity index, body shape index, and abdominal volume index.

**Results:**

Anthropometric measures were unrelated to overall ovarian cancer, high-grade serous cancer, and non-high-grade serous cancer. For example, the HR for overall ovarian cancer per standard deviation increment of body mass index at baseline was 0.98 (95% CI 0.88–1.10). Similar associations were observed with measurements of body fat distribution.

**Conclusion:**

These results do not indicate that adult adiposity is associated with ovarian cancer risk in post-menopausal women.

**Supplementary Information:**

The online version contains supplementary material available at 10.1007/s10552-020-01377-y.

## Introduction

Ovarian cancer is the fifth most common cause of cancer death in women in North America and western Europe [1]. Meta-analyses and pooled observational studies suggest that obesity may be positively related to ovarian cancer risk [2–5]. The World Cancer Research Fund/American Institute for Cancer Research concluded that the evidence for the link between obesity and increased ovarian cancer risk is probable [6], and an umbrella review graded the evidence as suggestive [3]. A growing body of research further suggests that body composition plays an important role in site-specific cancer development [7–9]. Previous studies indicate that anthropometric measures of general obesity and body fat distribution may be differentially related to ovarian cancer risk; however, results have been conflicting [4, 5, 10–12]. We conducted a cohort study among post-menopausal women using data from the National Institutes of Health-American Association of Retired Persons (NIH-AARP) Diet and Health Study to provide further insights into the association between body fatness and subtype-specific epithelial ovarian cancer risk by comparing indicators of general obesity and body fat distribution.

## Methods

### Study population

The NIH-AARP Diet and Health Study is a prospective cohort study of persons in the U.S. [13]. At baseline (1995–1996), 3.5 million AARP members aged 50–71 years who resided in six states (California, Florida, Louisiana, New Jersey, North Carolina, and Pennsylvania) and two metropolitan areas (Atlanta, Georgia; and Detroit, Michigan) were invited to complete a questionnaire on demographic, diet, and lifestyle characteristics. Questionnaires were completed satisfactorily by 566,398 participants. Six months after completing the baseline questionnaire, a second questionnaire was mailed to living participants who did not have a self-reported colon, breast, or prostate cancer at baseline to collect additional information. Self-reported weight and height were collected on the baseline questionnaire. Self-reported waist circumference and hip circumference were assessed on the second questionnaire. We excluded male participants (*n* = 339,666); those with unknown cancer or previous diagnosis of cancer other than non-melanoma skin cancer at baseline before completion of the second questionnaire (*n* = 16,300); those with bilateral oophorectomy and unknown oophorectomy status (*n* = 57,047); those with no information on height, weight, waist circumference, or hip circumference (*n* = 95,764); and subjects with body mass index less than 18.5 kg/m^2^ or more than 65 kg/m^2^ (*n* = 3,528). Our final analytical datasets included 145,575 women for the analysis on body mass index, 60,999 for waist circumference, 60,826 for hip circumference, 60,597 for waist-to-hip ratio, 60,999 for waist-to-height ratio, 60,826 for body adiposity index, 60,999 for body shape index, and 60,597 for abdominal adiposity index. The Special Studies Institutional Review Board of the U.S. National Cancer Institute approved the study [13]. All participants gave informed consent by virtue of completing and returning the baseline questionnaire.

### Assessment of anthropometric measures

Self-reported height and weight were obtained from the baseline questionnaire. Body mass index was calculated as weight (kg) divided by the square of height (in meters). In the second questionnaire, participants were instructed to measure their waist circumference and hip circumference using a tape measure to the nearest 0.25 inch while standing. Waist circumference was to be measured 1 inch above the navel if this was not the waistline. Hip circumference was defined as the largest circumference between the upper edge of the pelvis and the femur. Waist–hip ratio was calculated by dividing waist circumference (cm) by hip circumference (cm), and waist–height ratio was calculated as waist circumference (cm)/height (cm). The body adiposity index was calculated as ${\text{hip}}\;{\text{circumference}}\;{\text{(cm)}}/{\text{height}}^{{{1}{\text{.5}}}} \left( {\text{m}} \right) - {18}$ [14]. The body shape index was based on $\text{waist circumference (cm)}/{\text{body mass index}}^{\frac{2}{{3}}}\times {\text{height}}^{2}\left({\text{m}}\right)$ [15]. The abdominal volume index was also quantified [16]:

$$(2\;{\text{cm}} \times {\text{waist}}\;{\text{circumference}}^{2} \;({\text{cm}}) + 0.7\;{\text{cm}} \times ({\text{waist}}\;{\text{circumference}}\;({\text{cm}}) - {\text{hip}}\;{\text{circumference}}\;({\text{cm}}))^{2} )/1{,}000.$$

### Definition of cancer outcomes

Diagnoses of ovarian cancer were ascertained through 31 September 2011, via linkage to state cancer registries of the eight recruitment areas where the study participants were most likely to relocate: Arizona, Nevada, and Texas. This approach has been estimated to yield a sensitivity of 90% and a specificity of nearly 100% [17]. Newly diagnosed ovarian cancer cases were identified using the International Classification of Diseases for Oncology (ICD-O-3), topography (C56.9), and morphology codes (8441, 8460, 8461, 8470, 8471, 8480, 8481, 8380, 8381, 8560, 8570, 8310, 8313, 8010, 8020, 8021, 8050, 8070, 8120, 8140, 8255, 8260, 8323, 8440, 8450, 8562, 9000) [18]. The high-grade serous group included all invasive serous cancers except low grade [19, 20]. The non-high-grade group included all serous cancers and invasive mucinous, endometrial, and clear cell cancers [20]. Borderline tumors were excluded from this study.

### Baseline confounders

We controlled for several baseline participant characteristics that were assumed to cause adiposity or ovarian cancer [3, 5, 21–23]. We assumed that direct causes of the exposure or outcome, excluding possible instrumental variables, would identify a sufficient set of confounders [24]. Potential confounding variables included age, education (no school degree/unknown, primary school, technical school/secondary, university), participants’ self-reported information on race/ethnicity (none-Hispanic white, other), cigarette smoking (never, current < 15 cigarettes per day, current ≥ 15 cigarettes/day, former < 10 years, former ≥ 10 years), alcohol consumption (in grams of pure alcohol per day), parity (0, 1, ≥ 2 children), age at menarche (≤ 12 years, > 12 years), family history of ovarian cancer, oral contraceptive use, and menopausal hormone therapy.

### Statistical analysis

Cox proportional hazards regression was used for estimating adjusted hazard ratios (HRs) and 95% confidence intervals (CIs) for overall ovarian cancer and ovarian cancer subtypes. Time of study entry was age at baseline (or second questionnaire) and exit time was age at cancer diagnosis or the last date at which follow-up was considered complete. Athropometric measures were modeled as a continuous and categorical metric. After confirming that the linearity assumption was met by testing cubic spline transformations [25], HRs were estimated per standard deviation (1-SD) increase in anthropometric measures. Body mass index (kg/m^2^) was further grouped as normal weight (18.5 to < 25), overweight (25 to < 30), and obese (≥ 30). Waist circumference and other anthropometric measures were categorized according to quartiles. Models were stratified by 5-year age groups to minimize departure from proportionality and adjusted for education, race, smoking, alcohol consumption (modeled continuously using restricted cubic splines [25]), parity, age at menarche, family history of ovarian cancer, oral contraceptive use, and menopausal hormone therapy.

The improvement in predictive accuracy after adding anthropometric measures to a null model (including race, age, education, smoking, alcohol consumption, parity, family history of ovarian cancer, oral contraceptive use and menopausal hormone therapy) was evaluated in terms of explained variation (*R*^2^) [26], the Bayes Information Criterion (BIC), and model discrimination using Harrell’s C-index [25] derived from flexible parametric models [27]. *p* values for the difference between Harrell’s C indices of models with and without anthropometric indicators were computed using the method proposed by Antolini et al. [28]. We used 1,000 bootstrap replications to perform internal validations and to correct *R*^2^, BIC, and Harrell’s C-indices for optimism [25].

In a sensitivity analysis, we used regression calibration for self-reported body mass index, waist circumference, and hip circumference to assess possible regression dilution bias [29]. Because replicate measurements were not available, we applied published reliability coefficients [30–33], ranging from 0.5 to 0.9. A further threat to the validity of our estimates is potential unobserved confounding by undiagnosed ovarian cancer (often referred to as “reverse causation” [34]) if these conditions are symptomatic enough to induce a change in body weight. We, therefore, assumed 3 years of minimum latent period required for weight change due to unobserved disease to affect the outcome and excluded events that occurred during this time [35]. The statistical analysis was performed using Stata 15.1.

## Results

In the analytical sample of 145,575 women, the mean (SD) age at baseline was 61.8 (5.4) years. During a median follow-up time of 12.6 years, participants contributed 1,897,323 person-years and 683 ovarian cancer (343 high-grade serous) cases occurred. The baseline characteristics of the analytical sample are provided in Table 1.

**Table 1:** Baseline characteristics of ovarian cancer cases among 145,575 women in the NIH-AARP Study

	All study subjects	No ovarian cancer	Ovarian cancer	Histological subtypes	
				High-grade serous	Non-high-grade serous
Age (years)	61.8 (5.4)	61.8 (5.4)	62.7 (5.4)	62.4 (5.4)	61.9 (5.7)
Education (*n* (%))					
< 12 years	8,259 (5.6)	8,223 (5.6)	36 (5.1)	11 (3.1)	7 (4.8)
12 years	37,549 (25.6)	37,374 (26.6)	175 (24.9)	85 (24.2)	43 (29.4)
> 12 years	100,736 (68.7)	100,243 (68.7)	493 (70.0)	255 (72.7)	96 (65.8)
Race (*n* (%))					
Non-hispanic white	137,456 (91.2)	136,763 (91.3)	693 (94.93)	355 (96.2)	141 (94.6)
Non-hispanic black	7,883 (7.9)	7,864 (5.3)	19 (2.6)	4 (1.1)	6 (4.0)
Hispanic/other	5,199 (5.2)	5,181 (3.5)	18 (2.5)	10 (2.7)	2 (1.3)
Body mass index (kg/m^2^)	26.8 (5.5)	26.8 (5.5)	26.7 (5.5)	26.2 (4.8)	26.4 (5.2)
18.5 to < 25 (*n* (%))	67,266 (44.7)	66,936 (44.7)	330 (45.2)	179 (48.5)	64 (43.0)
25 to < 30 (*n* (%))	49,207 (32.7)	48,979 (32.2)	228 (31.2)	115 (31.2)	53 (35.6)
30+ (*n* (%))	34,065 (22.6)	33,893 (22.6)	172 (23.6)	75 (20.3)	32 (21.5)
Waist circumference (cm)	84.6 (13.2)	84.6 (13.2)	85.3 (13.2)	84.0 (11.6)	86.6 (15.8)
Quartile 1 (*n* (%))	15,346 (25.0)	15,268 (24.4)	78 (25.2)	40 (25.1)	16 (28.8)
Quartile 2 (*n* (%))	16,600 (25.0)	16,533 (26.5)	67 (21.6)	41 (25.8)	5 (8.9)
Quartile 3 (*n* (%))	15,618 (25.0)	15,538 (24.9))	80 (25.8)	41 (25.8)	17 (30.4)
Quartile 4 (*n* (%))	15,247 (25.0)	15,162 (24.3)	85 (27.4)	37 (23.3)	18 (32.1)
Hip circumference (cm)	103.9 (11.5)	103.9 (11.5)	104.5 (11.4)	103.4 (10.2)	104.3 (11.9)
Quartile 1 (*n* (%))	19,006 (25.0)	18,919 (30.4)	87 (28.1)	49 (31.0)	14 (25.0)
Quartile 2 (*n* (%))	13,197 (25.0)	13,128 (21.1)	69 (22.3)	37 (23.4)	11 (19.6)
Quartile 3 (*n* (%))	15,192 (25.0)	15,126 (24.3	66 (21.3)	33 (20.9)	12 (21.4)
Quartile 4 (*n* (%))	15,231 (25.0)	15,143 (24.3)	88 (28.4)	29 (24.7)	19 (33.9)
Waist–hip ratio	0.81 (0.08)	0.81 (0.08)	0.81 (0.08)	0.81 (0.07)	0.81 (0.09)
Quartile 1 (*n* (%))	15,525 (25.0)	15,454 (24.9)	71 (22.9)	35 (22.2)	17 (30.4)
Quartile 2 (*n* (%))	15,704 (25.0)	15,628 (25.2)	76 (24.6)	44 (27.9)	7 (12.5)
Quartile 3 (*n* (%))	15,833 (25.0)	15,755 (25.4)	78 (25.2)	40 (25.3)	13 (23.2)
Quartile 4 (*n* (%))	15,317 (25.0)	15,233 (24.5)	84 (27.2)	39 (24.7)	19 (33.9)
Waist–height ratio	0.52 (0.08)	0.51 (0.08)	0.52 (0.0.8)	0.51 (0.07)	0.52 (0.10)
Quartile 1 (*n* (%))	15,360 (25.0)	15,283 (24.5)	77 (24.8)	36 (22.6)	17 (30.4)
Quartile 2 (*n* (%))	16,183 (25.0)	16,116 (25.8)	67 (21.6)	40 (25.2)	7 (12.5)
Quartile 3 (*n* (%))	15,811 (25.0)	15,731 (25.2)	80 (25.8)	45 (28.3)	14 (25.0)
Quartile 4 (*n* (%))	15,457 (25.0)	15,371 (24.6)	86 (27.7)	38 (23.9)	18 (32.1)
Body adiposity index	31.9 (5.8)	31.8 (5.8)	32.2 (6.0)	31.7 (5.3)	32.1 (5.9)
Quartile 1 (*n* (%))	15,045 (25.0)	14,976 (24.0)	69 (22.3)	34 (21.5)	11 (19.6)
Quartile 2 (*n* (%))	16,882 (25.0)	16,800 (27.0))	82 (26.5)	51 (32.3)	11 (19.6)
Quartile 3 (*n* (%))	15,524 (25.0)	15,445 (24.8)	79 (25.5)	40 (25.3)	20 (35.7)
Quartile 4 (*n* (%))	15,175 (25.0)	15,095 (24.2)	80 (25.8)	33 (20.9)	14 (25.0)
Body shape index	0.01 (0.003)	0.01 (0.003)	0.01 (0.003)	0.01 (0.003)	0.01 (0.003)
Quartile 1 (*n* (%))	15,302 (25.0)	15,226 (24.4)	76 (24.5)	33 (20.8)	13 (23.2)
Quartile 2 (*n* (%))	15,958 (25.0)	15,884 (25.4)	74 (23.9)	36 (22.6)	17 (20.4)
Quartile 3 (*n* (%))	16,294 (25.0)	16,212 (25.9)	82 (26.5)	46 (28.9)	18 (32.1)
Quartile 4 (*n* (%))	15,257 (25.0)	15,179 (24.3)	78 (25.2)	44 (27.7)	8 (14.3)
Abdominal volume index	15.0 (4.7)	15.0 (4.7)	15.2 (4.6)	14.7 (3.9)	15.5 (5.4)
Quartile 1 (*n* (%))	15,317 (25.0)	15,237 (24.6)	80 (25.9)	41 (26.0)	16 (28.6)
Quartile 2 (*n* (%))	16,005 (25.0)	15,942 (25.7)	63 (20.4)	39 (24.7)	5 (8.9)
Quartile 3 (*n* (%))	15,711 (25.0)	15,632 (25.2)	79 (25.6)	39 (24.7)	17 (30.4)
Quartile 4 (*n* (%))	15,346 (25.0)	15,259 (25.6)	87 (28.2)	39 (24.7)	18 (32.1)
Smoking status (*n* (%))					
Never smoked	68,479 (45.5)	68,118 (45.5)	361 (49.5)	202 (54.7)	64 (42.9)
Former smoker, ≤ 20 cigarettes per day	41,527 (27.6)	41,323 (27.6)	204 (27.9)	87 (23.6)	56 (37.6)
Former smoker, > 20 cigarettes per day	18,884 (12.5	18,803 (12.6)	81 (11.1)	38 (10.5)	14 (9.4)
Current smoker, ≤ 20 cigarettes per day	15,774 (10.5	15,709 (10.5)	65 (8.8)	33 (8.9)	14 (9.4)
Current smoker, > 20 cigarettes per day	5,874 (3.9)	5,855 (3,9)	19 (2.6)	9 (2.4)	1 (0.7)
Alcohol consumption (g/day)	6.2 (17.3)	6.2 (17.3)	6.0 (14.7)	5.4 (9.8)	5.5 (13.4)
Parity (*n* (%))					
Never had a child	22,954 (15.3)	22,819 (5.3)	135 (18.5)	63 (17.1)	39 (26.4)
1 child	15,342 (10.2)	15,255 (10.3)	87 (11.9)	44 (11.9)	18 (12.2)
2 and more children	111,501 (74.4)	110,994 (74.4)	507 (69.4)	262 (70.0)	91 (51.5)
Age at menarche (*n* (%))					
≤ 12 years	72,734 (48.5)	72,375 (48.5)	359 (49.4)	191 (51.8)	67 (45.6)
> 12 years	77,150 (51.5)	76,782 (51.5)	368 (50.6)	178 (48.2)	80 (54.4)
Family history of ovarian cancer (*n* (%))	9,154 (6.1)	9,101 (6.1)	53 (7.3)	22 (5.9)	13 (8.7))
Ever oral contraceptive use (*n* (%))	60,739 (40.7)	60,500 (40.6)	239 (33.1)	115 (31.3)	64 (43.2)
Ever hormone replacement therapy (*n* (%))	69,739 (46.3)	69,370 (46.3)	369 (50.6)	201 (54.5)	70 (47.0)

NIH-AARP, NIH-AARP Diet and Health Study. Entries are means (standard deviation) for continuous variables and percent values for categorical variables

Neither body mass index nor other anthropometric measurements were associated with the risk of ovarian cancer (Table 2). For example, HRs for overall ovarian cancer per 1-SD increment in body mass index, waist circumference, hip circumference, waist–hip ratio, and waist–height ratio were 0.98 (95% CI 0.88–1.10), 1.08 (95% CI 0.91–1.27), 1.06 (95% CI 0.90–1.25), 1.03 (95% CI 0.89–1.21), and 1.08 (95% CI 0.92–1.27), respectively. In categorical analyses, the HRs for comparing obese and normal weight body mass index groups, and the highest and lowest quartiles of waist circumference, hip circumference, waist–hip ratio, and waist–height ratio were 1.11 (95% CI 0.91–1.36), 1.17 (95% CI 0.84–1.62), 1.28 (95% CI 0.94–1.75), 1.35 (95% CI 0.97; 1.89), and 1.18 (95% CI 0.85–1.64), respectively. No associations were observed for high-grade serous carcinomas. The accuracy of models predicting ovarian cancer risk was not improved after adding anthropometric measures (Table 3).

**Table 2:** Association of general obesity and indicators of body fat distribution with ovarian cancer in NIH-AARP

	Ovarian cancer risk	High-grade serous	Non-high-grade serous
Body mass index (*n* = 145,575), number of cases	683	343	141
HR per SD (95% CI)	0.98 (0.88; 1.10)	0.91 (0.77; 1.08)	0.92 (0.71; 1.19)
HR, categorical			
18.5 to < 25 (*n* (%))	Reference	Reference	Reference
25 to < 30 (*n* (%))	0.97 (0.81; 1.16)	0.94 (0.73; 1.20)	1.13 (0.77; 1.65)
≥ 30 (*n* (%))	1.11 (0.91; 1.36)	0.91 (0.68; 1.22)	1.03 (0.65; 1.63)
Joint *p* value	0.418	0.772	0.814
Waist circumference (*n* = 60,999), number of cases	295	151	54
HR per SD (95% CI)	1.08 (0.91; 1.27)	1.00 (0.79; 1.27)	1.10 (0.76; 1.60)
HR, categorical			
Quartile 1	Reference	Reference	Reference
Quartile 2	0.82 (0.59; 1.16)	1.09 (0.69; 1.70)	0.30 (0.11; 0.81)
Quartile 3	1.06 (0.76; 1.46)	1.16 (0.74; 1.84)	0.97 (0.48; 1.99)
Quartile 4	1.17 (0.84; 1.62)	1.07 (0.66; 1.73)	1.22 (0.61; 2.47)
Joint *p* value	0.226	0.932	0.099
Hip circumference (*n* = 60,826), number of cases	295	150	54
HR per SD (95% CI)	1.06 (0.90; 1.25)	0.98 (0.77; 1.24)	1.02 (0.70; 1.49)
HR, categorical			
Quartile 1	Reference	Reference	Reference
Quartile 2	1.07 (0.77; 1.48)	1.07 (0.69; 1.66)	1.13 (0.51; 2.50)
Quartile 3	0.91 (0.65; 1.27)	0.83 (0.52; 1.31)	1.00 (0.45; 2.20)
Quartile 4	1.28 (0.94; 1.75)	1.06 (0.68; 1.65)	1.67 (0.81; 3.43)
Joint *p* value	0.203	0.712	0.448
Waist–hip ratio (*n* = 60,597), number of cases	294	150	54
HR per SD (95% CI)	1.03 (0.89; 1.21)	1.03 (0.82; 1.29)	1.02 (0.70; 1.49)
HR, categorical			
Quartile 1	Reference	Reference	Reference
Quartile 2	1.16 (0.83; 1.63)	1.44 (0.91; 2.28)	0.38 (0.15; 0.98)
Quartile 3	1.15 (0.82; 1.61)	1.22 (0.75; 1.97)	0.84 (0.40; 1.76)
Quartile 4	1.35 (0.97; 1.89)	1.38 (0.85; 2.24)	1.31 (0.66; 2.61)
Joint *p* value	0.362	0.435	0.069
Waist–height ratio (*n* = 60,999), number of cases	295	151	54
HR per SD (95% CI)	1.08 (0.92; 1.27)	1.00 (0.79; 1.27)	1.13 (0.78; 1.64)
HR, categorical			
Quartile 1	Reference	Reference	Reference
Quartile 2	0.86 (0.61; 1.20)	1.22 (0.76; 1.94	0.35 (0.14; 0.89)
Quartile 3	1.06 (0.76; 1.47)	1.42 (0.89; 2.25)	0.80 (0.38; 1.67)
Quartile 4	1.18 (0.85; 1.64)	1.22 (0.74; 2.00)	1.16 (0.58; 2.33)
Joint *p* value	0.304	0.530	0.080
Body adiposity index (*n* = 60,826), number of cases	294	150	54
HR per SD (95% CI)	1.07 (0.91; 1.26)	0.98 (0.77; 1.25)	1.06 (0.76; 1.55)
HR, categorical			
Quartile 1	Reference	Reference	Reference
Quartile 2	1.00 (0.72; 1.39)	1.33 (0.85; 2.08)	0.92 (0.40; 2.13)
Quartile 3	1.06 (0.76; 1.48)	1.13 (0.70; 1.82)	1.78 (0.84; 3.77)
Quartile 4	1.14 (0.81; 1.48)	0.99 (0.59; 1.65)	1.27 (0.55; 2.93)
Joint *p* value	0.850	0.508	0.279
Body shape index (*n* = 60,999), number of cases	294	151	54
HR per SD (95% CI)	0.98 (0.83; 1.16)	1.08 (0.85; 1.36)	0.82 (0.55; 1.21)
HR, categorical			
Quartile 1	Reference	Reference	Reference
Quartile 2	0.84 (0.60; 1.17)	0.92 (0.56; 1.50)	1.16 (0.55; 2.44)
Quartile 3	0.94 (0.68; 1.30)	1.18 (0.74; 1.88)	1.17 (0.56; 2.46)
Quartile 4	0.93 (0.67; 1.30)	1.20 (0.75; 1.93)	0.56 (0.23; 1.40)
Joint *p* value	0.787	0.611	0.342
Abdominal volume index (*n* = 60,597), number of cases	295	150	
HR per SD (95% CI)	1.07 (0.91;1.25)	0.98 (0.77; 1.25)	1.13 (0.79; 1.61)
HR, categorical			
Quartile 1	Reference	Reference	Reference
Quartile 2	0.78 (0.55; 1.10)	1.04 (0.66; 1.63)	0.31 (0.11; 0.84)
Quartile 3	1.00 (0.73; 1.39)	1.06 (0.67; 1.68)	0.96 (0.47; 1.97)
Quartile 4	1.15 (0.83; 1.59)	1.09 (0.68; 1.74)	1.21 (0.60; 2.44)
Joint *p* value	0.155	0.988	0.061

NIH-AARP, NIH-AARP Diet and Health Study. HR (hazard ratio) from age-group stratified multivariable Cox model adjusted for education, race, smoking, alcohol consumption, parity, age at menarche, family history of ovarian cancer, oral contraceptive use, and menopausal hormone therapy. Joint *p* value from Wald test of all exposure dummy variables

**Table 3:** General obesity and indicators of body fat distribution for prediction of ovarian cancer

	Baseline model	Body mass index	Waist circumference	Hip circumference	Waist-to-hip ratio	Waist-to-height ratio	Body adiposity index	Body shape index	Abdominal volume index
Ovarian cancer									
Adjusted *R*^2^	0.059	0.059	0.071	0.068	0.066	0.072	0.068	0.069	0.068
BIC	16,349.94	16,361.70	6,650.949	6,629.188	6,627.817	6,650.704	6,628.799	6,652.352	6,626.746
Harrell’s C-index (*p*)	0.60	0.60 (0.599)	0.62 (0.389)	0.62 (0.333)	0.62 (0.473)	0.62 (0.342)	0.62 (0.241)	0.62 (0.310)	0.62 (0.395)
High-grade serous									
Adjusted *R*^2^	0.099	0.100	0.1337	0.126	0.127	0.134	0.135	0.112	0.126
BIC	8,284.92	8,295.007	3,481.144	3,460.403	3,459.172	3,481.147	3,480.754	3,491.962	3,459.309
Harrell’s C-index (*p*)	0.63	0.63 (0.149)	0.66 (0.175)	0.66 (0.193)	0.66 (0.188)	0.66 (0.174)	0.66 (0.178)	0.65 (0.256)	0.66 (0.189)
Non-high-grade serous									
Adjusted *R*^2^	0.089	0.089	0.064	0.069	0.064	0.062	0.053	0.060	0.061
BIC	3,547.038	16,361.7	1,385.031	1,385.028	1,373.11	1,384.8	1,383.999	1,385.7	1,383.915
Harrell’s C-index (*p*)	0.65	0.66 (0.643)	0.64 (0.213)	0.64 (0.193)	0.64 (0.164)	0.64 (0.186)	0.65 (0.094)	0.65 (0.124)	0.65 (0.208)

Null model included the predictor’s age, education, race, smoking, alcohol consumption, physical activity, parity, family history of ovarian cancer, family history of breast cancer, hormone therapy. Adjusted *R*^2^: explained variation

BIC: Bayes Information Criterion. *p*: *p* value for difference of Harrell’s C vs null model

Sensitivity analysis indicated that HRs could have been attenuated towards the null because of measurement error in self-reported anthropometric measurements. For example, the unadjusted HR for per SD of waist circumference and overall ovarian cancer was 1.08 (95% CI 0.91–1.27), but after accounting for potential regression dilution bias, assuming an attenuation factor of 0.7, the HR was 1.11 (95% CI 0.91–1.30) (Supplementary Table 1). The associations were virtually unchanged when events occurring during the first three years of follow-up were excluded (Supplementary Table 2).

## Discussion

This study examined the association of indicators of general obesity and body fat distribution with ovarian cancer risk using a large U.S. prospective cohort study of post-menopausal women. We found no association of anthropometric measures with the risk of overall ovarian cancer, high-grade serous, or non-high-grade ovarian cancers. Overall, our analysis does not support the hypothesis that central adiposity or measures of body fat distribution improve the prediction of ovarian cancer risk in post-menopausal women.

A larger body of research examined the association between obesity and ovarian cancer risk. However, the findings of more than 30 epidemiologic studies have been weak and mixed [36]. Several meta-analyses and pooled analyses reported weak positive associations between adult body mass index and ovarian cancer risk, noting substantial between-study heterogeneity with weaker associations in prospective than case–control studies [2, 6, 37]. A systematic review from the World Cancer Research Fund and the American Institute for Cancer Research included 28 prospective studies on ovarian cancer and calculated a summary relative risk for a 5-unit increment in body mass index of 1.07 (95% CI 1.03–1.11) [2, 6]. The effect size was similar in the post-menopausal group but it was less precise (relative risk per 5 kg m^−2^ = 1.07; 95% CI 1.00–1.14). Results from the 2013 Ovarian Cancer Association Consortium [4] pooled analysis of case–control studies found that the positive association with body mass index was stronger in pre-menopausal women. The heterogeneity of findings reported here and previously could be explained by menopausal status and higher statistical efficacy of meta-analysis. Indeed, our HRs for body mass index and waist circumference overlap with the HR’s CIs of the World Cancer Research Fund/American Institute for Cancer Research meta-analysis [2, 6], which indicates that there might be chance variation of estimates. Few studies have examined how different measures of body fat distribution are related to ovarian cancer and its subtypes [10–12]. Existing cohort studies found no association of waist circumference and waist–hip ratio and ovarian cancer risk [2, 6, 10]. Similar to previous studies [4, 5], the present study found no notable differences between histotypes. In contrast, a Mendelian randomization study suggested that obesity might cause non-high-grade but not high-grade ovarian cancer [20].

The present study has several limitations. It relied on self-reported anthropometric data and potential measurement error could have attenuated the observed associations. Anthropometric measurements were taken when most study participants had reached menopause. However, pre-menopausal anthropometric risk factors might be more strongly related to ovarian cancer risk. A Mendelian randomization study suggested a positive association with higher body mass index with risk of ovarian cancer among pre-menopausal women, but not for post-menopausal women [38]. Unfortunately, we could not test whether the association between anthropometric markers and ovarian cancer risk was modified by menopause status. More studies on body weight and body fatness changes over the lifecourse are warranted [39, 40]. Our study also lacked updated information on anthropometric measurements during follow-up. Another drawback is the low number of cases by ovarian cancer subtype and a lack of statistical power to test for effect modification.

In summary, results from this prospective study of post-menopausal women do not support associations between measures of central obesity and body fat distribution and risk of ovarian cancer.

## Supplementary Information

Below is the link to the electronic supplementary material.

Supplementary file1 (DOCX 29 KB)

## Data Availability

Data are available upon request from https://www.aarp.org/forms/research-dataset-request-form/.

## Code availability

Code is available upon request from the corresponding author.
