# Supplementary material for: Anthropometric risk factors for ovarian cancer in the NIH-AARP Diet and Health Study
Source: Cancer Causes Control. 2021 Jan 22;32(3):231–9. doi: 10.1007/s10552-020-01377-y (PMC7870624; doi:10.1007/s10552-020-01377-y)
Supplement: Supplementary file 1 — Supplementary file1 (DOCX 29 KB) [file 10552_2020_1377_MOESM1_ESM.docx]

**Anthropometric risk factors for ovarian cancer in the NIH-AARP Diet and Health Study**

Sebastian E Baumeister et al.

Supplement

Supplementary Table 1 Association of general Obesity, indicators of body fat distribution and ovarian cancer in NIH-AARP using regression calibration to examine possible regression dilution bias

|  | Ovarian Cancer Risk | High-grade serous | Non-high grade serous |
| --- | --- | --- | --- |
|  | HR^1^ per SD (95% CI) | HR^1^ per SD (95% CI) | HR^1^ per SD (95% CI) |
| **Body mass index** |  |  |  |
| λ=0.5 | 0.96 (0.82; 1.11) | 0.84 (0.65; 1.02) | 0.85 (0.53; 1.16) |
| λ=0.7 | 0.97 (0.87; 1.08) | 0.88 (0.74; 1.02) | 0.89 (0.65; 1.13) |
| λ=0.9 | 0.98 (0.90; 1.06) | 0.91 (0.79; 1.02) | 0.91 (0.72; 1,11) |
| **Waist circumference** |  |  |  |
| λ=0.5 | 1.16 (0.88; 1.44) | 1.01 (0.72; 1.30) | 1.21 (0.32; 2.11) |
| λ=0.7 | 1.11 (0.91; 1.30) | 1.00 (0.80;1.21) | 1.15 (0.54; 1.76) |
| λ=0.9 | 1.08 (0.94; 1.23) | 1.00 (0.84;1.16) | 1.11 (0.65; 1.57) |
| **Hip circumference** |  |  |  |
| λ=0.5 | 1.13 (0.84; 1.42) | 0.95 (0.63;1.27) | 1.04 (0.51; 1.57) |
| λ=0.7 | 1.09 (0.89; 1.29) | 0.97 (0.73;1.20) | 1.03 (0.66; 1.39) |
| λ=0.9 | 1.07 (0.92; 1.22) | 0.97 (0.79;1.15) | 1.03 (0.66; 1.39) |

^1^HR (hazard ratio) adjusted for attenuation factor (regression dilution ratio) λ: ${HR}^{1/\lambda}$ with bootstrapped 95% confidence intervals (CI). Age-group stratified multivariable Cox model adjusted for education, race, smoking, alcohol consumption, parity, age at menarche, family history of ovarian cancer, oral contraceptive use, and menopausal hormone therapy.

Appendix Table 2 Association of General obesity, indicators of body fat distribution and ovarian cancer in NIH-AARP excluding the first three years of follow-up to reduce bias due to reverse causation

|  | Ovarian Cancer Risk | High-grade serous | Non-high grade serous |
| --- | --- | --- | --- |
|  |  |  |  |
| **Body mass index***,* number of cases | 481 | 242 | 93 |
| HR per SD (95% CI) | 0.99 (0.86; 1.13) | 0.90 (0.73; 1.10 | 0.93 (0.68; 1.28) |
| HR, categorical |  |  |  |
| 18.5 to <25 (n (%)) | Reference | Reference | Reference |
| 25 to <30 (n (%)) | 0.96 (0.77; 1.18) | 0.93 (0.70; 1.25) | 1.08 (0.67; 1.72) |
| 30+ (n (%)) | 1.14 (0.90; 1.45) | 0.89 (0.63; 1.27) | 1.06 (0.61; 1.86) |
| Joint *P*-value | 0.351 | 0.793 | 0.949 |
| **Waist circumference***,* number of cases | 199 | 105 | 35 |
| HR per SD (95% CI) | 1.10 (0.90; 1.34) | 1.02 (0.77; 1.35) | 0.99 (0.60; 1.61) |
| HR, categorical |  |  |  |
| Quartile 1 | Reference | Reference | Reference |
| Quartile 2 | 0.75 (0.50; 1.14) | 1.13 (0.66; 1.96) | 0.15 (0.03; 0.66) |
| Quartile 3 | 1.05 (0.71; 1.55) | 1.35 (0.78; 2.32) | 0.67 (0.27; 1.64) |
| Quartile 4 | 1.21 (0.82; 1.79) | 1.11 (0.62; 2.00) | 1.13 (0.50; 2.58) |
| Joint *P*-value | 0.155 | 0.747 | 0.050 |
| **Hip circumference***,* number of cases | 198 | 104 | 35 |
| HR per SD (95% CI) | 1.02 (0.83; 1.24) | 0.95 (0.71; 1.28) | 0.87 (0.52; 1.45) |
| HR, categorical |  |  |  |
| Quartile 1 | Reference | Reference | Reference |
| Quartile 2 | 0.90 (0.60; 1.34) | 0.92 (0.53; 1.59) | 0.78 (0.31; 1.96) |
| Quartile 3 | 0.76 (0.51; 1.14) | 0.95 (0.56; 1.61) | 0.30 (0.09; 1.06 |
| Quartile 4 | 1.21 (0.84; 1.75) | 1.00 (0.59; 1.71) | 1.30 (0.58; 2.94) |
| Joint *P*-value | 0.148 | 0.987 | 0.145 |
| **Waist-hip ratio***,* number of cases | 199 | 104 | 35 |
| HR per SD (95% CI) | 1.09 (0.93; 1.28) | 1.07 (0.84; 1.36) | 1.08 (0.71; 1.64) |
| HR, categorical |  |  |  |
| Quartile 1 | Reference | Reference | Reference |
| Quartile 2 | 1.11 (73; 1.70) | 1.28 (0.73; 2.25) | 0.47 (0.16; 1.36) |
| Quartile 3 | 1.26 (0.83; 1.90) | 1.36 (0.77; 2.39) | 0.59 (0.22; 1.60) |
| Quartile 4 | 1.60 (1.07; 2.40) | 1.45 (0.82; 2.58) | 1.43 (0.62; 3.32) |
| Joint *P*-value | 0.104 | 0.615 | 0.113 |
| **Waist-height ratio***,* number of cases | 199 | 105 | 35 |
| HR per SD (95% CI) | 1.10 (0.93; 1.28) | 1.01 (0.76; 1.34) | 1.02 (0.63; 1.67) |
| HR, categorical |  |  |  |
| Quartile 1 | Reference | Reference | Reference |
| Quartile 2 | 0.82 (0.54; 1.25) | 1.17 (0.66; 2.08) | 0.34 (0.11; 1.04 |
| Quartile 3 | 1.02 (0.68; 1.52) | 1.50 (0.86; 2.61) | 0.73 (0.29; 1.82) |
| Quartile 4 | 1.33 (0.90; 1.97) | 1.35 (0.75; 2.43) | 1.12 (0.47; 2.66) |
| Joint *P*-value | 0.129 | 0.520 | 0.190 |
| **Body adiposity index***,* number of cases | 198 | 104 | 35 |
| HR per SD (95% CI) | 1.02 (0.84; 1.25) | 0.96 (0.72; 1.28) | 0.93 (0.56; 1.55) |
| HR, categorical |  |  |  |
| Quartile 1 | Reference | Reference | Reference |
| Quartile 2 | 1.03 (0.69; 1.48) | 1.70 (0.97; 2.96) | 0.64 (0.24; 1.69) |
| Quartile 3 | 0.98 (0.64; 1.48) | 1.31 (0.72; 2.39) | 0.93 (0.37; 2.32) |
| Quartile 4 | 1.19 (0.79; 1.79) | 1.18 (0.62; 2.21) | 1.03 (0.40; 2.64) |
| Joint *P*-value | 0.773 | 0.251 | 0.779 |
| **Body shape index***,* number of cases | 199 | 105 | 35 |
| HR per SD (95% CI) | 1.04 (0.85; 1.28) | 1.14 (0.86; 1.51) | 0.93 (0.56; 1.55) |
| HR, categorical |  |  |  |
| Quartile 1 | Reference | Reference | Reference |
| Quartile 2 | 0.77 (0.51; 1.17) | 0.80 (0.44; 1.46) | 1.52 (0.55; 4.68) |
| Quartile 3 | 1.00 (0.67; 1.48) | 1.21 (0.80; 2.10) | 1.71 (0.63; 4.68) |
| Quartile 4 | 1.06 (0.71; 1.58) | 1.30 (0.74; 2.28) | 1.06 (0.34; 3.26) |
| Joint *P*-value | 0.451 | 0.347 | 0.627 |
| **Abdominal volume index***,* number of cases | 198 | 104 | 35 |
| HR per SD (95% CI) | 1.09 (0.90;1.32) | 0.99 (0.74; 1.32) | 1.03 (0.63; 1.68) |
| HR, categorical |  |  |  |
| Quartile 1 | Reference | Reference | Reference |
| Quartile 2 | 0.69 (0.45; 1.06) | 1.04 (0.60; 1.81) | 0.15 (0.03; 0.68) |
| Quartile 3 | 0.96 (0.65; 1.42) | 1.19 (0.69; 2.05) | 0.66 (0.27; 1.62) |
| Quartile 4 | 1.19 (0.81; 1.75) | 1.14 (0.64; 2.02) | 1.12 (0.49; 2.54) |
| Joint *P*-value | 0.088 | 0.924 | 0.057 |

NIH-AARP, NIH-AARP Diet and Health Study. HR (hazard ratio) from age-group stratified multivariable Cox model adjusted for education, race, smoking, alcohol consumption, parity, age at menarche, family history of ovarian cancer, oral contraceptive use, and menopausal hormone therapy. Joint P-value from a Wald test of all exposure dummy variables
